# Downregulation of metallothionein-2 contributes to oxaliplatin-induced neuropathic pain

**DOI:** 10.1186/s12974-021-02139-6

**Published:** 2021-04-13

**Authors:** Xuelin Huang, Jie Deng, Ting Xu, Wenjun Xin, Yuehong Zhang, Xiangcai Ruan

**Affiliations:** 1 Department of Anesthesia and Pain Medicine, Guangzhou First People’s Hospital, School of Medicine, South China University of Technology, Guangzhou, 510000 Guangdong China; 2 Guangdong Province Key Laboratory of Brain Function and Disease, Department of Physiology, Zhongshan School of Medicine, Sun Yat-Sen University, Guangzhou, 510000 China; 3 Department of Ophthalmology, Guangzhou First People’s Hospital, School of Medicine, South China University of Technology, Guangzhou, 510000 Guangdong China; 4 Department of Ophthalmology, The Second Affiliated Hospital of South China University of Technology, 602 Renminbei Road, Guangzhou, 510180 China; 5 Department of Anesthesia and Pain Medicine, The Second Affiliated Hospital of South China University of Technology, 602 Renminbei Road, Guangzhou, 510180 China

**Keywords:** Chemotherapy-induced peripheral neuropathy, Metallothionein-2, Oxaliplatin, Mechanical allodynia, NF-κB

## Abstract

**Background:**

We previously reported a correlation between small doses of oxaliplatin penetrating onto the spinal cord and acute pain after chemotherapy. Here, we propose that MT2 within the spinal dorsal horns participates in the development of oxaliplatin-induced neuropathic pain and may be a pharmacological target for the prevention and treatment of chemotherapy-induced peripheral neuropathy (CIPN).

**Methods:**

The rat model of CIPN was established by 5 consecutive injections of oxaliplatin (0.4 mg/100 g/day). Genetic restoration of neuron-specific metallothionein-2 was implemented 21 days before oxaliplatin treatment, and also, genetic inhibition by metallothionein-2 siRNA was performed. Mechanical allodynia and locomotor activity were assayed. Cell-specific expression of metallothionein-2, the mRNA levels of pro-inflammatory cytokines, nuclear translocation of NF-κB, the protein levels of expression of IκB-α, and interaction between IκB-α and P65 were evaluated in the spinal dorsal horns. Also, in vitro interaction of sequentially deleted IκB-α promoter with metallothionein-2 was used to assess the signal transduction mechanism.

**Results:**

We found that oxaliplatin induced downregulation of metallothionein-2 in rat spinal cord neurons. By contrast, genetic restoration of metallothionein-2 in the spinal dorsal horn neuron blocked and reversed neuropathic pain in oxaliplatin-treated rats of both sexes, whereas genetic inhibition of metallothionein-2 triggered neuropathic pain in normal rats. Overall locomotor activity was not impaired after the genetic alterations of metallothionein-2. At the molecular level, metallothionein-2 modulated oxaliplatin-induced neuroinflammation, activation of NF-κB, and inactive transcriptional expression of IκB-α promoter, and these processes could be blocked by genetic restoration of metallothionein-2 in the spinal dorsal horn neurons.

**Conclusions:**

Metallothionein-2 is a potential target for the prevention and treatment of CIPN. A reduction of NF-κB activation and inflammatory responses by enhancing the transcription of IκB-α promoter is proposed in the mechanism.

## Introduction

Oxaliplatin, a third-generation platinum-based antitumor drug, is commonly used to treat many types of solid tumors [1, 2]. However, its anticancer efficacy is accompanied by serious side effects, including chemotherapy-induced peripheral neuropathy (CIPN) [3]. More than 90% of all cancer patients treated with oxaliplatin experience acute, transient sensory disturbances in the distal extremities. Although these usually resolve within a few days, 20–54% of treated patients complain of persistent neuropathy with chronic pain [3–6]. This painful oxaliplatin-induced peripheral neuropathy can last up to 5 years beyond the end of the chemotherapy [7] and has a deleterious impact on the quality of life of cancer survivors and patients under treatment [8, 9]. Currently, the mechanism of CIPN is not fully defined, and a chemotherapy dose reduction or early discontinuation compromising effective cancer treatment is often the sole choice for its relief or prevention [10, 11].

In a previous study, we demonstrated a correlation between the penetration of small doses of oxaliplatin into the spinal cord and acute pain after systemic oxaliplatin administration [12]. Other laboratories have also reported complementary findings on the direct central nerve system (CNS) effects of chemotherapeutic paclitaxel [13, 14], and neuropathological changes in the spinal dorsal horn neurons have therefore gained significant interest [15, 16]. Like the peripheral neuropathological changes that occur in dorsal root ganglion neurons and distal nerve endings, the central effects of very low concentrations of chemotherapeutics are associated with the development of overt CIPN [8, 17]. Also, like the well-documented peripheral effects, no clear preventative treatment exists, and the underlying mechanisms need further investigation.

One possible mechanism might involve metallothioneins (MTs), a family of low-molecular-weight proteins that are actively involved in alterations in gene transcription, detoxification of toxic metals, anti-inflammation, and protection against oxidative stress [18, 19]. Recent studies have demonstrated that MT1/2 proteins are expressed in healthy nerves but not in painful neuroma nerves [20], and this expression may protect against the development of inflammatory and neuropathic pain [21]. MT2, encoded by the MT2A gene, has emerged as a key player in the processes of neuroprotection and neuroregeneration [22, 23]. MT2 can regulate the cell inflammatory responses through inactivation of the NF-κB pathway and inhibits the activation of pro-inflammatory cytokines, including TNF-α [19]. A negative association has also been shown between the expression of MT1/2 and neuroinflammation in the brain [24]. Importantly, MT2 appears capable of reducing gastric and hepatocellular tumorigenicity [25, 26], so targeting MT2 will not interfere with the anti-cancer efficacy of chemotherapeutics. However, a role for MT2 dysregulation in the development of painful CIPN remains to be established.

The hypothesis tested in the present study is that MT2 participates in the painful CNS effects of oxaliplatin and may be a pharmacological target for the prevention and treatment of oxaliplatin-associated neurotoxicity.

## Materials and methods

### Animals

All experimental procedures and protocols used in this study were approved by the local Animal Care Committee and conformed to the National Institutes of Health Guide for the Care and Use of Laboratory Animals. Male and female Sprague-Dawley rats weighing 200–220 g were obtained from the Institute of Experimental Animals of Sun Yat-Sen University and used throughout this study. The animals were distributed into different experimental or control conditions randomly in the present study. All animals were housed in separate cages at 23 ± 3°C, at 50 to 60% humidity and a 12-h/12-h light-dark cycle, and were provided with food and water ad libitum.

### Drug administration

Oxaliplatin (Aladdin Reagent Co., Shanghai, China) was dissolved in 5% glucose solution to a concentration of 1mg/mL. Rats were intraperitoneally (i.p.) injected at 0.4 mg/100 g once per day for 5 consecutive days; control animals were injected with an equivalent volume of the 5% glucose vehicle.

Recombinant AAV-hSyn-MT2A-EGFP (AAV-MT2A-EGFP) and its AAV-EGFP control were designed and constructed by standard methods by BrainVTA (Wuhan, China). Briefly, 150 nL of AAV-MT2A-EGFP or AAV-EGFP was injected intraspinally into both sides of the L5 spinal dorsal horn of naive rats at 4 injection sites. Oxaliplatin and its vehicle were consecutively administered 21 days after the AAV virus injection [12, 27]. MT2A siRNA or non-targeting control siRNA was designed and purchased from Ribobio Technology (Guangzhou, China). Intrathecal (i.t.) injection of MT2A siRNA or control siRNA (3 nmol, 10 μL) was performed in naive rats once every 3 days for a total of three injections.

### Behavior assessment

#### Mechanical allodynia assessment

von Frey hairs were used to test mechanical allodynia [27]. Briefly, each animal was placed individually in a transparent plastic box and allowed to adapt to the environment for three consecutive days (15 min/day) before behavior tests. Baseline behavioral measurements were taken to assess mechanical allodynia before the administration of test substances or their vehicles. Different strengths of von Frey fibers were used alternately to stimulate the mid-plantar surface of the hind paw. If no paw withdrawal reaction occurred, the next stronger fiber was selected for stimulation; if a paw withdrawal reaction occurred, the weaker stimulus was chosen. The absolute paw withdrawal threshold (g) was measured as the force that caused the rat to withdraw its paw. Testing was only performed in the morning hours. The “up and down” method was used to define the mechanical sensitivity that produced a 50% likelihood of paw withdrawal. The values obtained from both hind paws were averaged.

#### Locomotor activity assessment

An open field was used to assess overall locomotor activity level. The rats were first placed in the center of a 100×100 cm box. After a 1-min habituation to the box environment, the locomotor activity was recorded for a total of 5 min. The DigBehv software was used to analyze the total distance traveled while the rat was in the box. This total distance was defined as the overall locomotor activity level.

All behavioral tests were conducted by a researcher who was blind to the treatment conditions.

### RNA extraction and real-time quantitative PCR

The rats were euthanized with sodium pentobarbital at a dose of 100 mg/kg (i.p.). The spinal cord was immediately removed, and the L4–L6 segment of the spinal dorsal horn was isolated and stored at − 80 °C. Total RNA was extracted from the spinal cord tissues using TRIzol Reagent (Invitrogen, USA). The subsequent reverse transcription was performed according to the protocol provided with the polymerase chain reaction (PCR) kit (Takara, Shiga, Japan). The cDNA was amplified with specific primers (Table 1) to investigate the mRNA. The qPCR reaction was conducted at 95 °C for 30 s, followed by a thermal cycle of 5 s at 95 °C, 30 s at 60 °C (repeated for 40 cycles), and final stabilization at 95 °C. The expression ratio of mRNA in the spinal dorsal horn was normalized to β-actin and analyzed by the 2^−ΔΔCT^ method.

**Table 1:** Rat specific primer sequences for the genes used

Gene	Primer	Sequence
MT2A	Forward	5′-TGCAAATGCACCTCCTGCAA-3′
	Reverse	5′-GCACTTGTCCGAAGCCTCTT-3′
IL-1β	Forward	5′-TGTTTCCCTCCCTGCCTCTGAC-3′
	Reverse	5′-CGACAATGCTGCCTCGTGACC-3′
IL-6	Forward	5′-GAGACTTCCAGCCAGTTGCC-3′
	Reverse	5′-ACTGGTCTGTTGTGGGTGGTA-3′
TNF-α	Forward	5′-AGCACGGAAAGCATGATCCG-3′
	Reverse	5′-TGAGAAGAGGCTGAGGCACA-3′
β-Actin	Forward	5′-GGAGATTACTGCCCTGGCTCCTA-3′
	Reverse	5′-GACTCATCGTACTCCTGCTTGCTG-3′

### Western blotting

Rats were sacrificed, and the L4–L6 segment of the spinal dorsal horn was quickly isolated and stored at − 80 °C. The dorsal horn proteins were extracted with lysis buffer (Beyotime, P0013, Shanghai, China), a protease cocktail, and phosphatase inhibitors. The protein samples were separated by SDS-PAGE and then transferred onto a PVDF membrane. After blocking in block buffer for 1 h at room temperature, the PVDF membrane was incubated overnight at 4 °C with primary antibodies against MT2A (1:1000, Abcam), IκB-α (1:2000, Abcam), P65 (1:5000, Abcam), and GAPDH (1:1000, CST). The blots were then incubated for 1 h at room temperature with the appropriate secondary antibodies. The antigen-antibody complexes were visualized by enhanced chemiluminescence (Pierce), and the selected bands were quantified using a computer-assisted imaging analysis system (NIH ImageJ).

### Enzyme-linked immunosorbent assay (ELISA)

The dorsal horn tissues were homogenized with ice-cold PBS and centrifuged at 5000*g* for 15min, and then the supernatant was collected. The levels of IL-1β, TNF-α, and IL-6 were analyzed with the ELISA Assay Kit (MEIMIAN, Jiangsu, China, MM-0047R, MM-0190R, MM-0180R) according to the manufacturer’s instructions.

### Extraction of nuclear and cytoplasmic proteins

The nuclear and cytoplasmic proteins isolated from the L4–L6 segment of the spinal dorsal horn were extracted using the NE-PER nuclear and cytoplasmic extraction reagents kit (Thermo, 78833) according to the manufacturer’s instructions. Finally, the nuclear and cytoplasmic extracts were analyzed separately by western blot.

### Immunohistochemistry

Rats were anesthetized deeply with sodium pentobarbital (50 mg/kg, i.p.) and perfused through the ascending aorta with 4% paraformaldehyde before dissection of the L4–L6 segment of the spinal dorsal horn. The segment tissue was placed into 4% paraformaldehyde for post-fixing overnight, followed by dehydration in 30% sucrose for two nights, and the tissue was then cut transversely into consecutive sections 25-μm-thick. After blocking in block buffer for 1 h at room temperature, the sectioned tissues were incubated overnight at 4 °C with primary antibody for MT2A (1:250, Abcam), NeuN (1:200, Chemicon), GFAP (1:200, Chemicon), and Iba1 (1:200, Chemicon), followed by incubation with secondary antibody for 1 h at room temperature. The blocking buffer for the immunocytochemistry was 5% normal donkey serum in 0.01M PBS with 0.3% Triton X-100. The immunofluorescence of the stained slices was then examined with a Nikon (Nikon, Italy) fluorescence microscope, and images were captured with a Nikon Eclipse Ni-E camera for further analysis.

### Coimmunoprecipitation assays (Co-IP)

Coimmunoprecipitation assays were performed with a co-immunoprecipitation kit (Pierce, 88804) according to the manufacturer’s instructions, as previously described [28]. In brief, the spinal dorsal horn tissues were quickly minced and homogenized in lysis buffer. The extracted protein (300 μg) was incubated at 4 °C overnight with IκB-α antibody (1:50, Abcam), and then 20 μL magnetic beads (Millipore, Boston, MA) were added to each sample. The samples were incubated on a 4 °C rotary shaker for 4 h, washed with lysis buffer, and then heated to 96 °C for 10 min with 20 μL of loading buffer to elute the immunocomplexes. The immunocomplexes were then analyzed by western blot as we described.

### Dual-luciferase reporter assays

Luciferase reporter and expression plasmids were co-transfected into 293T cells cultured in 24-well plates. After 48 h, the luciferase activity of the cells was measured using the Duo-Luciferase Assay kit (FulenGen, Guangzhou, China). The results were calculated based on the relative ratio of Renilla versus firefly luciferase activity before being compared with control vectors.

### Statistical analysis

SPSS 26.0 was used to analyze all the data, which were expressed as means ± SEM. The Shapiro-Wilk test was used to determine normality. One-way ANOVA, Student’s *t* test, or the Kruskal-Wallis test (for non-normally distributed data) were used to analyze the histological, western blotting, and qPCR data. Post hoc analyses were performed using Tukey’s honestly significant difference or Dunn’s multiple comparisons test, as appropriate. Behavioral tests were analyzed using the rank sum test when tests of normality were not satisfied. No statistical methods were used to predetermine the sample sizes, but the sample sizes are similar to those reported in previous studies of painful behavior and pertinent molecular studies [12, 27]. In all cases, a two-tailed *p* < 0.05 was considered statistically significant.

## Results

### Oxaliplatin downregulated MT2 in the spinal dorsal horn neurons of male rats

**Fig. 1:**
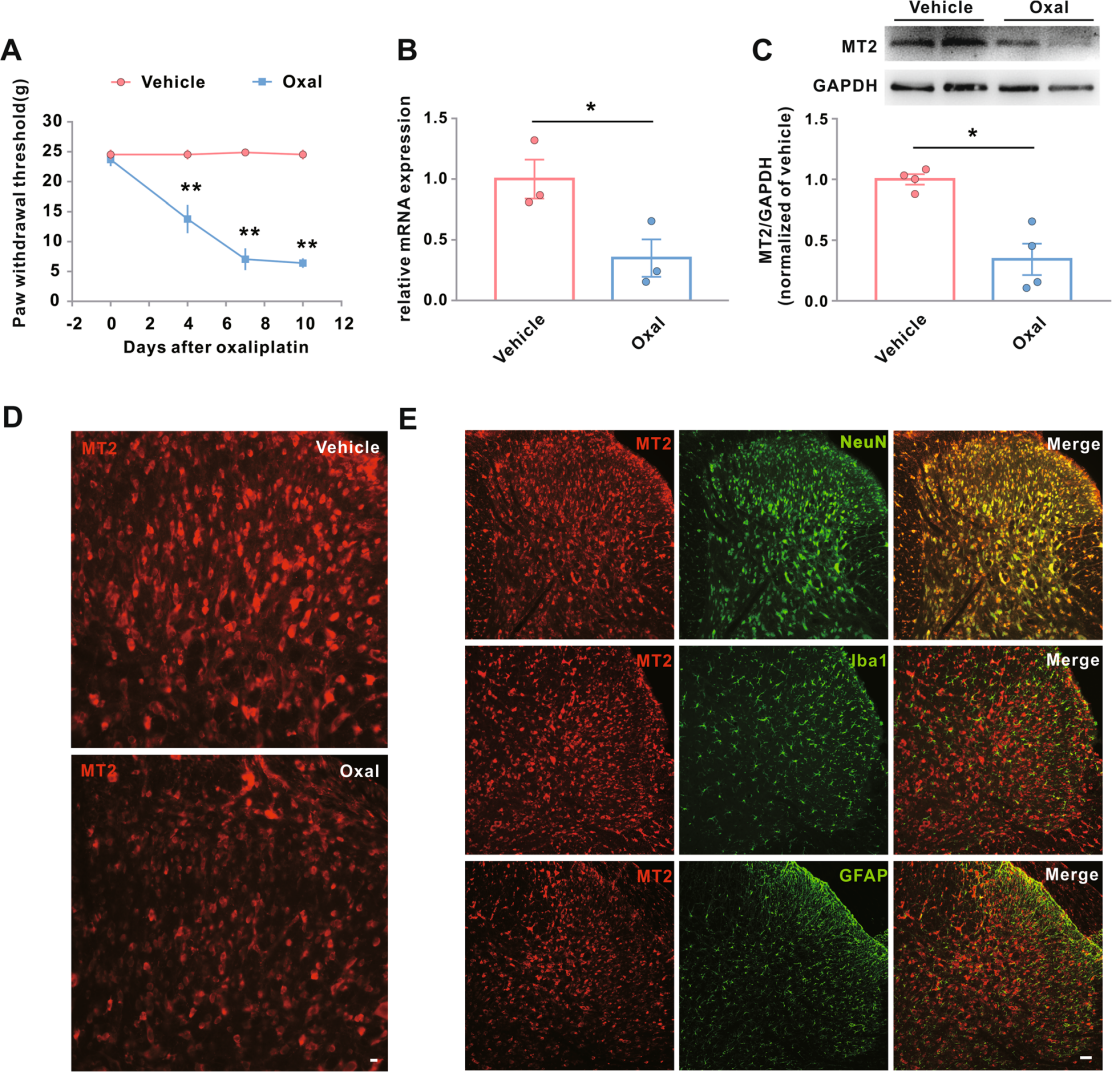
Downregulation of MT2 in spinal dorsal horn neurons is associated with Oxal-induced neuropathic pain. Male rats were treated with vehicle or Oxal (0.4 mg/100 g/day for five consecutive days). **a** Hind paw withdrawal threshold response to von Frey filament stimuli on days 0, 4, 7, and 10 after the treatment. **p < 0.01 versus vehicle, *n* = 8. **b**–**e** Expression of MT2 in the L4–L6 spinal dorsal horn 10 days after the treatment. *p < 0.05, *n* = 4. **b** Transcriptional levels of MT2. **c** Translational levels of MT2. **d** Immunostaining for MT2. Scale bar, 100 μm. **e** Double immunostaining for MT2 (red) with NeuN (neuron marker, green), GFAP (astrocyte marker, green), or Iba1 (microglia marker, green). Scale bar, 50 μm. Oxal, oxaliplatin

We demonstrated the role of MT2 in CIPN by first examining the cell-specific MT2 abundance in the spinal cord in conjunction with the behavioral responses in male rats after consecutive administrations of oxaliplatin. Consistent with our previous studies [12], a 5-day administration of oxaliplatin, but not vehicle, produced a robust mechanical hypersensitivity, as evidenced by marked decreases in the mechanical withdrawal threshold over several time points (Fig. 1a). A significant decrease from baseline occurred beginning on day 4 compared with the vehicle-treated rats, and the pain hypersensitivities persisted until the end of the observation period (day 10).

The transcriptional and translational expression levels of MT2 in the dorsal horn samples were downregulated at day 10 after oxaliplatin treatment when compared to rats administered vehicle only (Fig. 1b, c). The downregulated MT2 expression was also identified by immunohistochemical staining (Fig. 1d). Examination of the distribution patterns of MT2 expression in the L4–L6 spinal dorsal horn on day 10 after oxaliplatin treatment revealed that MT2 was colocalized with NeuN (a neuronal marker), but not with Iba1 (a microglial marker) or GFAP (an astrocyte marker) (Fig. 1e). Collectively, these data showed an association between the downregulation of MT2 in the spinal dorsal horn neurons and oxaliplatin-induced neuropathic pain, indicating a potential role for MT2 in the development of CIPN.

### Impact of MT2 on oxaliplatin-induced neuropathic pain in both sexes

**Fig. 2:**
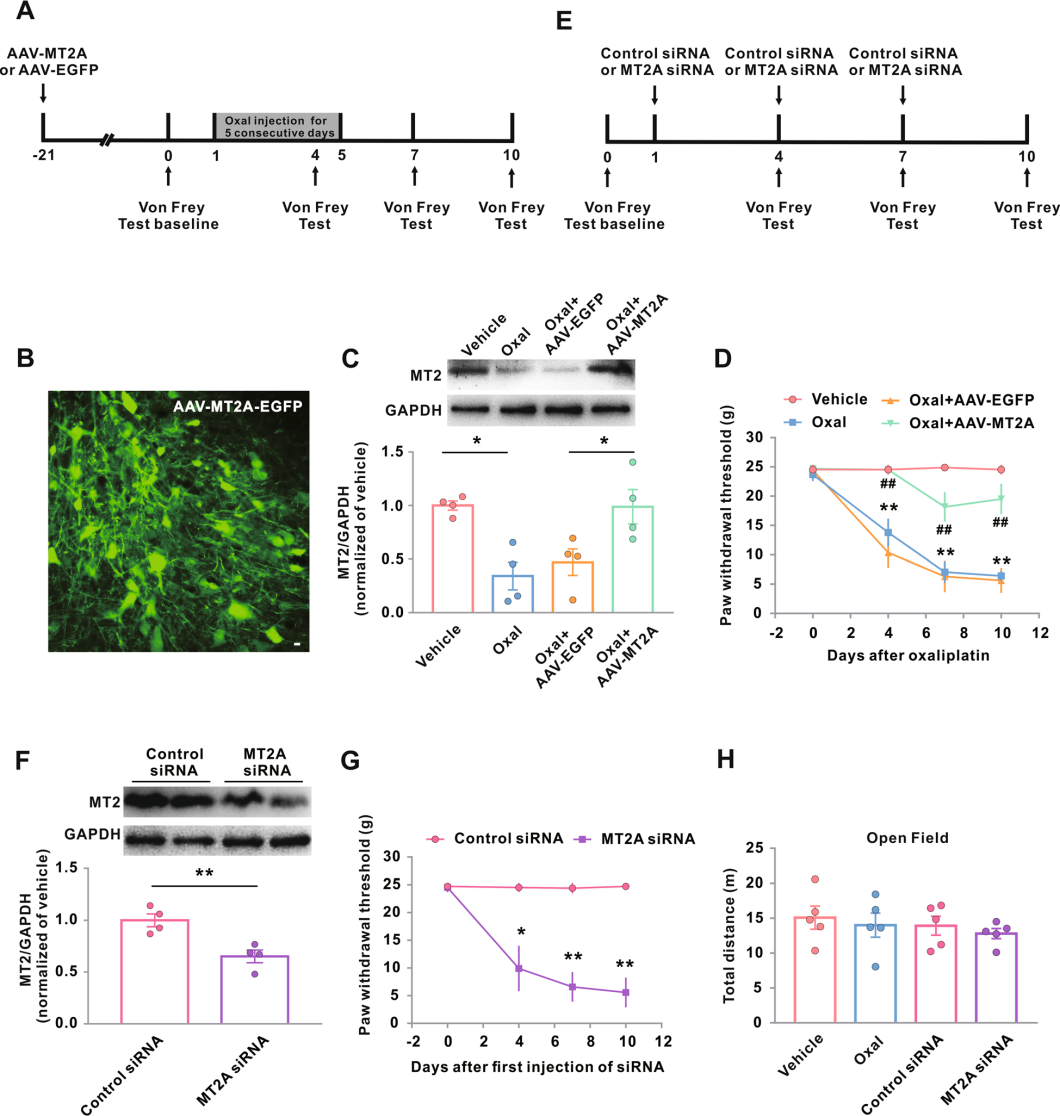
Impact of genetic modification of MT2 on Oxal-induced neuropathic pain. **a**–**e** Experimental timeline for **b**–**d**, **f**–**h**, Fig. 3, and Fig. 5. The arrows above the timeline mark the injection of AAV-EGFP-MT2A or AAV-EGFP (**a**) or mark the injection of MT2A siRNA or control siRNA (**e**); the arrows below the timeline mark the von Frey test time points. The administration of vehicle or Oxal (0.4 mg/100 g/day for five consecutive days) was carried out 21 days after intraspinal injection of AAV-MT2A-EGFP in male rats. **b** Neuron-specific green fluorescence in the L5 spinal dorsal horn in rats 21 days after intraspinal injection of AAV-MT2A-EGFP. Scale bar, 100 μm. **c** MT2 protein expression in the groups as indicated. Representative western blots (top panels) and a summary of densitometric analysis (bottom graphs). *p < 0.05; *n* = 4. **d** Hind paw withdrawal threshold response to von Frey filament stimuli on days 0, 4, 7, and 10 after the treatment. **p < 0.01 versus vehicle, ^##^*p* < 0.01 versus Oxal + AAV-EGFP; *n* = 8 in Oxal + AAV-EGFP; *n* = 11 in Oxal + AAV-MT2A-EGFP. **f** Intrathecal injection of MT2A siRNA reduced protein expression of MT2A. **p < 0.01; *n* = 4. **g** Hind paw withdrawal threshold in normal male rats. *p < 0.05 and **p < 0.01 versus control siRNA; *n* = 6. **h** Oxaliplatin administration or MT2A siRNA did not show reduced locomotor activity 10 days after oxaliplatin or vehicle treatment (*n* = 5). Oxal, oxaliplatin

We examined whether MT2 in the spinal dorsal horn neurons participated in the development of CIPN by rescuing the oxaliplatin-induced downregulation of MT2 by injecting a constructed AAV-MT2A-EGFP into the L5 spinal dorsal horn (Fig. 2a). We first tested the specificity and efficiency of microinjection of AAV-MT2A-EGFP into the spinal dorsal horn of naive rats. Twenty-one days after the microinjection, green fluorescent label was observed in the restricted dorsal horn neurons, indicating a high specificity and efficiency of the transfection (Fig. 2b). We then examined the impact of AAV-MT2A-EGFP microinjection on the behavioral responses in male rats after oxaliplatin administration. As expected, AAV-MT2A-EGFP rescued the oxaliplatin-induced decrease in the protein levels of MT2 in the spinal dorsal horn, compared with AAV-EGFP control (Fig. 2c). Importantly, the restoration of MT2 also significantly attenuated the decreases in the mechanical withdrawal threshold (Fig. 2d).

Pharmacologic tools are not yet available for MT2 intervention, so we next examined the effect of genetic inhibition of MT2 on the mechanical withdrawal threshold by intrathecal injection of MT2A siRNA (Fig. 2e). Naive rats injected with MT2A siRNA, when compared with rats injected with control siRNA, showed dramatic decreases in the translational levels of MT2 (Fig. 2f). Consistently, microinjection of MT2A siRNA also significantly decreased the mechanical withdrawal threshold in naive rats (Fig. 2g).

We also evaluated whether MT2 had adverse effects on the overall locomotor activity functions using open field tests. The rats given oxaliplatin or vehicle treatments showed no significant differences in total distances traveled in the box. Injection with MT2A siRNA also did not produce any significant differences in the locomotor distance when compared with control siRNA (Fig. 2h), indicating that MT2 intervention may not affect the overall locomotor activity level.

**Fig. 3:**
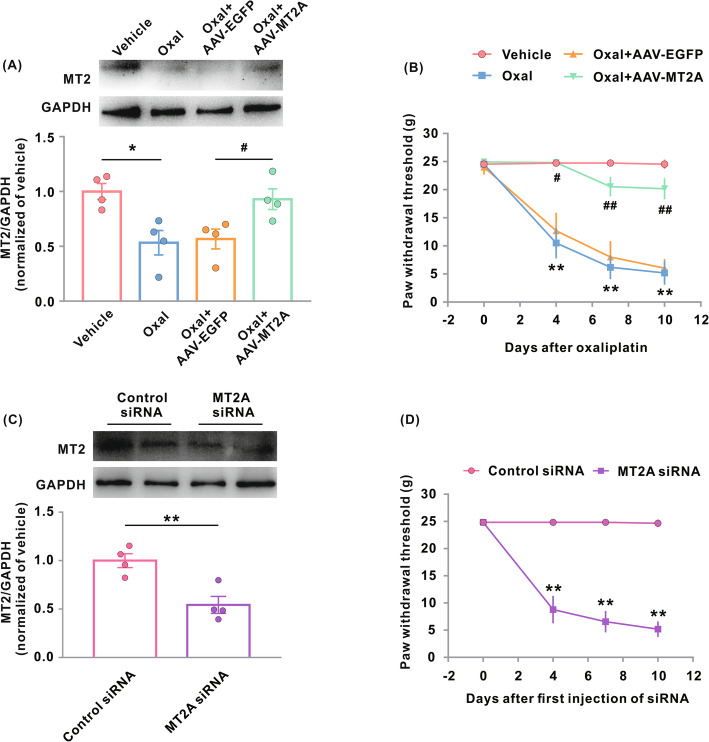
Impact of genetic modification of MT2 on Oxal-induced neuropathic pain in female rats. **a** MT2 protein expression in the spinal dorsal horn 10 days after the treatments. Representative western blots (top panels) and a summary of densitometric analysis (bottom graphs). *p < 0.05; *n* = 4. Female rats were treated with vehicle or Oxal (0.4 mg/100 g/day for five consecutive days) 21 days after intraspinal injection of AAV-MT2A-EGFP or AAV-EGFP. **b** Hind paw withdrawal threshold in the female rats treated in **a**. **p < 0.01 versus vehicle; ^#^*p* < 0.05, ^##^*p* < 0.01 versus Oxal + AAV-EGFP; *n* = 6 in Oxal + AAV-EGFP, *n* = 9 in Oxal + AAV-MT2A-EGFP. **c** MT2 protein expression after intrathecal injection of MT2A or control siRNA in female rats. Representative western blots (top panels) and a summary of densitometric analysis (bottom graphs). **p < 0.01; *n* = 4. **d** Hind paw withdrawal threshold in female rats treated with intrathecal injection of MT2A or control siRNA. **p < 0.01 versus control siRNA; *n* = 7. Oxal, oxaliplatin

Many studies have shown that certain pain pathways may vary considerably between male and female rats [29–31]. Therefore, we also examined the role of MT2 in oxaliplatin-treated female rats. Oxaliplatin induced MT2 downregulation and mechanical allodynia of similar magnitudes in the female rats to those seen in the male rats (Fig. 3a, b). Similarly, the restoration of MT2 in the spinal dorsal horn neurons prevented and reversed oxaliplatin-induced mechanical allodynia at similar magnitudes in both sexes (Fig. 3b). Microinjection of MT2A siRNA also produced similar mechanical allodynia in the female rats to that seen in the male rats (Fig. 3c, d).

Taken together, these findings suggest that MT2 in the spinal dorsal horn neurons plays a critical role in the development of oxaliplatin-induced neuropathic pain in both sexes. Since no significant differences were observed in the effect of MT2 modification on the oxaliplatin-induced pain behavior between male and female rats, we did not further investigate the effects in female rats in subsequent experiments.

### Oxaliplatin activates the NF-κB pathway and neuroinflammation

**Fig. 4:**
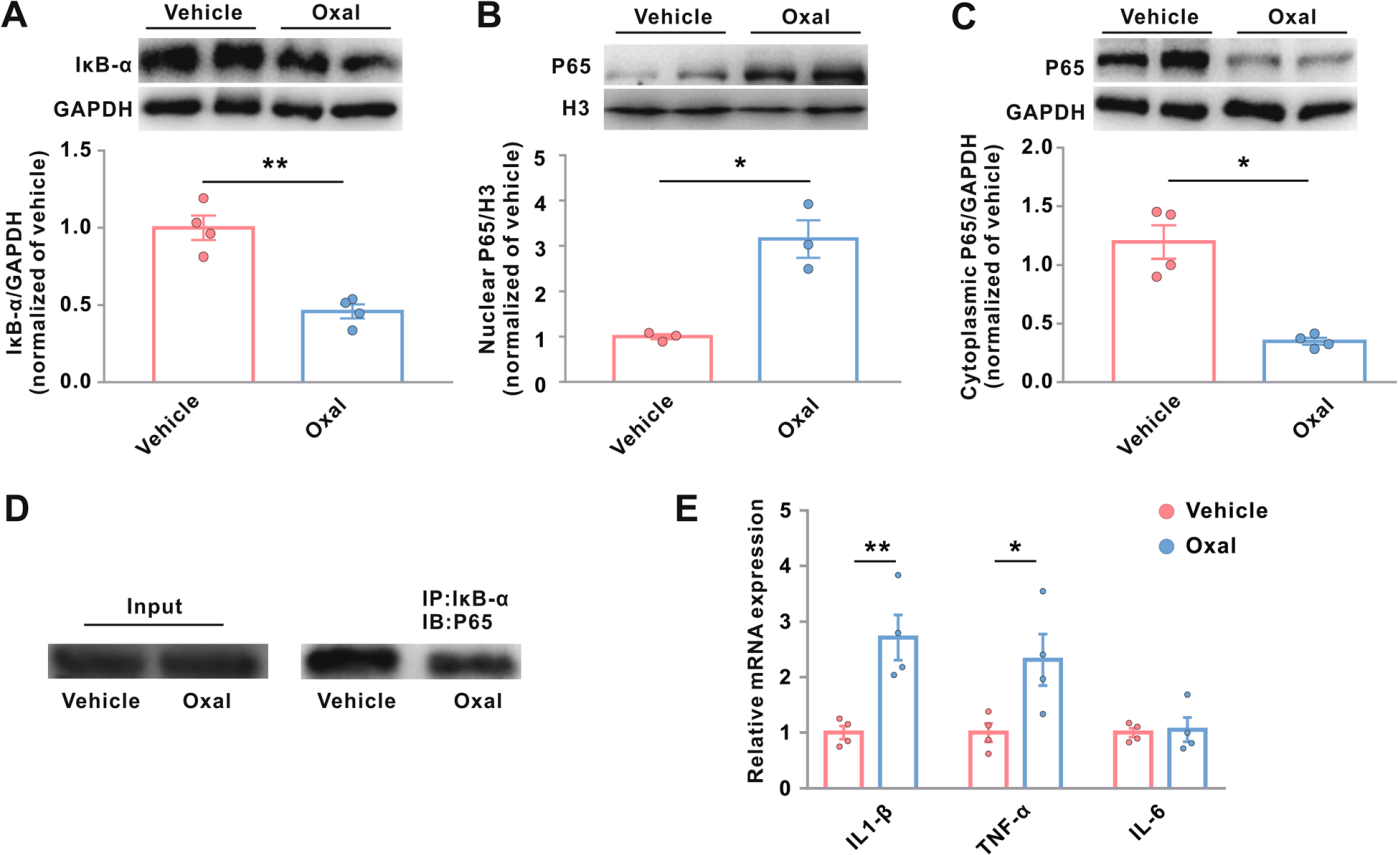
Oxaliplatin activates the NF-κB signaling pathway in the spinal dorsal horn. Rats were treated with vehicle or oxaliplatin (0.4 mg/100 g/day for five consecutive days), and the L4–L6 dorsal horns were harvested for the following examines. **a** Protein levels of IκB-α. GAPDH served as loading controls. **p < 0.01; *n* = 4. Protein levels of NF-κB P65 in the nucleus (**b**) and in the cytoplasm (**c**). H3 and GAPDH served as loading controls for nuclear and cytoplasmic proteins. *p < 0.05; *n* = 4. **d** IP with IκB-α antibody followed by immunoblotting using P65 antibody. **e** Transcriptional levels of IL-1β, TNF-α, and IL-6. *p < 0.05 and **p < 0.01; *n* = 4. Oxal, oxaliplatin

Previous studies have shown that activation of the NF-κB signaling pathway contributes to the acute pain induced by oxaliplatin [15]. Therefore, NF-κB signaling could be inferred to play an important role in the development of oxaliplatin-induced neuropathic pain. We tested this possibility by performing western blotting assays to assess the pathway alterations occurring in the spinal dorsal horn 10 days after oxaliplatin treatment. The spinal dorsal horn NF-κB inhibitor alpha (IκB-α) levels were downregulated by oxaliplatin treatment (Fig. 4a), and oxaliplatin administration also consistently induced translocation of the NF-κB p65 subunit from the cytoplasm into the nucleus (Fig. 4b, c). Immunoprecipitation assays showed that the interaction between IκB-α and P65 decreased after oxaliplatin treatment, confirming that oxaliplatin induced an activation of the NF-κB signaling pathway in the spinal dorsal horn (Fig. 4d).

We also evaluated the regulation of inflammatory responses and pro-inflammatory factors after oxaliplatin administration. Oxaliplatin administration upregulated the mRNA expression of IL-1β and TNF-α in the spinal dorsal horn, but it had no effect on the mRNA expression of IL-6 (Fig. 4e). Taken together, these data indicate that oxaliplatin activates the NF-κB signaling pathway to promote neuroinflammation in the spinal dorsal cord.

### MT2A inhibits oxaliplatin-induced NF-κB activation and neuroinflammation

**Fig. 5:**
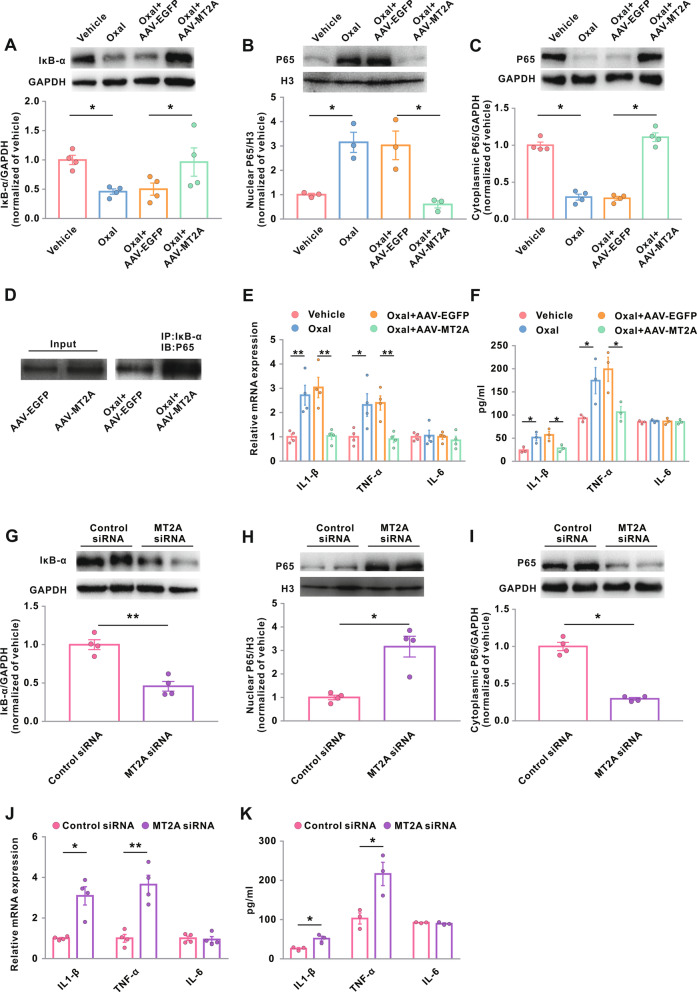
MT2 inhibits oxaliplatin-induced activation of the NF-κB signaling pathway in the spinal dorsal horn. **a**–**f** Rats were treated with vehicle or Oxal (0.4 mg/100 g/day for five consecutive days) 21 days after intraspinal injection of AAV-MT2A-EGFP or AAV-EGFP. **g**–**k** Naive rats were treated with intrathecal injection of MT2A or control siRNA. Then, the L4–L6 dorsal horns were harvested for the following examines. **a**, **g** Protein levels of IκB-α. GAPDH served as loading controls. *p < 0.05 and **p < 0.01; *n* = 4. Protein levels of NF-κB P65 in the nucleus (**b**, **h**) and in the cytoplasm (**c**, **i**). H3 and GAPDH served as loading controls for nuclear and cytoplasmic proteins, respectively. *p < 0.05; *n* = 4. **d** IP with IκB-α antibody followed by immunoblotting using P65 antibody. **e**, **j** Transcriptional levels of IL-1β, TNF-α, and IL-6. *p < 0.05 and **p < 0.01; *n* = 4. **f**, **k** Translational levels of IL-1β, TNF-α, and IL-6 in ELISA. *p < 0.05; *n* = 3. Oxal, oxaliplatin

Previous studies have reported that MT2 inhibits NF-κB activation in a number of disease models [26]. In the present study, AAV-hSyn-MT2A-EGFP, which was recognized to induce a genetic restoration of neuronal MT2, was intraspinally injected into oxaliplatin-treated rats to assess the subsequent expression of IκB-α and nuclear translocation of NF-κB p65. We found that genetic restoration of MT2 in the spinal dorsal horn neurons dramatically enhanced the expression of IκB-α and inhibited the nuclear translocation of p65 in oxaliplatin-treated rats (Fig. 5a–c). Our immunoprecipitation assays showed that genetic restoration of MT2 also rescued the oxaliplatin-inhibited interaction between IκB-α and P65 (Fig. 5d). Genetic restoration of MT2 prevented the oxaliplatin-induced upregulation of IL-1β and TNF-α in the spinal dorsal horn, with no effects on IL-6 expression, in both transcriptional and translational levels (Fig. 5e, f).

We also examined whether genetic inhibition of MT2 affected the activation of the NF-κB signaling pathway in normal animals. We found that administration of intrathecal MT2A siRNA promoted the degradation of IκB-α, enhanced the nuclear translocation of p65, and upregulated the transcriptional and translational expression of IL-1β and TNF-α in the spinal dorsal horn in the normal rats (Fig. 5g–k). Taken together, these data suggest that MT2 inhibits the NF-κB signaling pathway, thereby preventing oxaliplatin-induced neuroinflammation.

### MT2 enhances the transcription of IκB-α promoter in the spinal dorsal horn

**Fig. 6:**
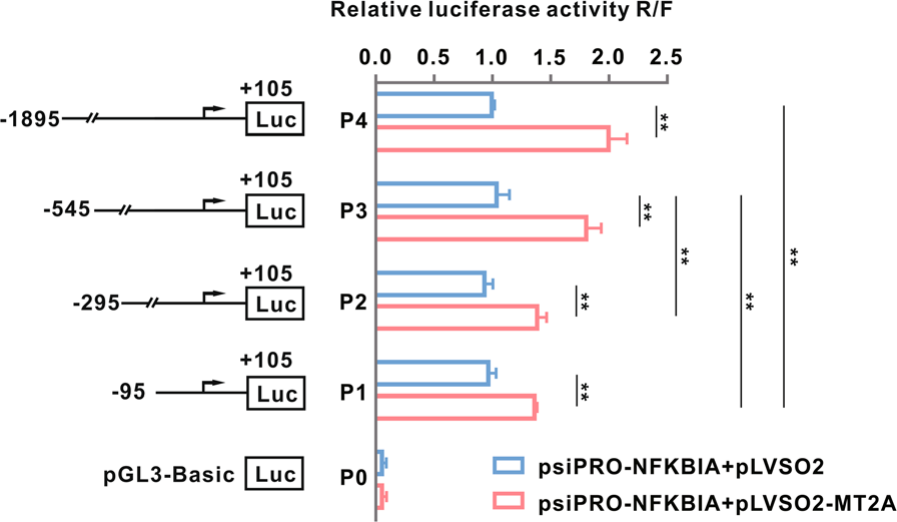
MT2A regulates IκB-α promoter transcriptionally to promote its expression. Luciferase reporter psiPRO-IκB-α (constructs with IκB-α promoter region from P1, −95 to +105; P2, −295 to +105; P3, −545 to +105; P4, −1895 to +105 of transcription start site [TSS], respectively) was co-transfected with pLVSO2-MT2A or control vector into 293T cells. **p < 0.01; *n* = 3

Several molecules inhibit the activation of NF-κB by maintaining a high level of its inhibitor, IκB-α, in the cytoplasm and thereby preventing the nuclear translocation of p65. Among these molecules, MT2 has been suggested to bind to the IκB-α promoter to block IκB-α degradation [32]. We confirmed this binding by constructing a luciferase reporter psiPRO-IκB-α with the IκB-α promoter region from −95 to +105, or −295 to +105, or −545 to +105, or −1895 to +105 of the transcription start site [TSS]. Cotransfection of the reporter with pLVSO2-MT2A or with a control vector into 293T cells (Fig. 6) showed that pLVSO2-MT2A induced a significant increase in the luciferase activity, indicating an interaction between MT2 and the IκB-α promoter. Specifically, the binding site is likely located in the −95 to +105 and −545 to −295 regions of the IκB-α promoter (Fig. 6). These data further support the notion that an interaction between MT2 and the IκB-α promoter regulates the NF-κB signaling pathway.

## Discussion

In the present work, we investigated the mediating role of MT2 in the development of painful CIPN. This is the first demonstration that downregulation of MT2 in the spinal dorsal horn neurons is involved in oxaliplatin-induced neuropathic pain. Mimicking this downregulation led to mechanical allodynia in rats, even in the absence of oxaliplatin treatment. Interestingly, the effects of MT2 modification on the oxaliplatin-induced pain behavior were similar between female and male rats, indicating that MT2 is a potential pharmacological target that will show no sex-gender differences in the management of painful CIPN.

At the molecular level, restoration of MT2 prevented the nuclear translocation of NF-κB while enhancing the interaction between IκB-α and P65. These molecular responses were consistent with the typical pain behavior observed in oxaliplatin-treated rats due to NF-κB activation and pro-inflammatory cytokine production. Neuroinflammation is an inflammatory response driven by pro-inflammatory cytokines that involves neurons and microglia in the spinal dorsal cord and is critical for the development of neuropathic pain [33]. Increases in inflammatory cytokines, notably IL-1β and TNF-α in the present study, contribute to the central sensitization and pain behavior associated with neuropathic pain [34].

Previous studies have reported that MT2 was capable of inhibiting the activation of pro-inflammatory cytokines, such as IL-12 and TNF-α and that their inhibition by MT2 contributed to recovery from neurodegenerative diseases [35]. We now show that genetic restoration of MT2 inhibited the expression of IL-1β and TNF-α in the spinal cord and attenuated the pain behavior induced by oxaliplatin. Consistent with our findings with MT2 restoration, we found that genetic inhibition of MT2 by intrathecal injection of MT2A siRNA in turn induced mechanical allodynia in normal mice through a similar neuroinflammatory signaling pathway. Our data are in line with previous reports showing a significant relationship between MT2 expression and cell inflammatory responses that act by suppressing NF-κB signaling activation [32, 36]. Therefore, MT2 is likely to contribute to oxaliplatin-induced neuropathic pain through the inactivation of the NF-κB signaling pathway in the spinal dorsal horn.

The re-expression of MT2 may prevent oxaliplatin-induced activation of NF-κB by triggering IκB-α gene transcription in the spinal dorsal horn. Lin et al. recently demonstrated that MT2 binds to the IκB-α promoter to regulate its transcription [32]. Our in vitro experiments showed that transfection of luciferase plasmids constructed with the IκB-α promoter region from −95 to +105 and −545 to −295 of TSS resulted in significant increases in the luciferase activity in 293T cells, but only when the cells were cotransfected with pLVSO2-MT2A. Therefore, the downregulation of MT2 induced by oxaliplatin in the spinal dorsal horn likely activates the NF-κB pathway by directly inhibiting IκB-α transcription. The interaction between IκB-α and MT2 regulation, especially under CIPN conditions, merits further study based on these intriguing data.

We also defined the main cells that express MT2 in the spinal cord during the development of CIPN. Double immunostaining showed intense intracellular staining *colocalized* with the neuron marker NeuN in the spinal dorsal horn, suggesting that MT2 is almost exclusively expressed in the spinal horn neurons after oxaliplatin treatment. These data indicate a potential role for MT2 in the development of CIPN, and this possibility is not surprising given the evident involvement of MT2 in different physiological and protective CNS processes [22, 24, 35]. Fortunately, the overall locomotor activity was not impaired in rats after either consecutive administration of oxaliplatin or genetic inhibition of MT2 via intrathecal siRNA injection. Some contrary results have been reported indicating that neurons appear to express the MT isoforms I and II to a much lower extent than is observed in astrocytes, and especially in the reactive astrocytes in the spinal cord and brain [37]. This discrepancy has since been resolved by the demonstration that neurons do upregulate the MTI/II expression in response to injury [38, 39]. More work is required to define the cell-specific roles of MT2 in the spinal cord during the development of painful CIPN.

Our findings in this study were limited to the neuropathological effects in the spinal dorsal horn as they have been reported to be exposed to very low concentrations of chemotherapeutics after systemic administration [12–14]. Downregulation of MT2 in the spinal dorsal horn neurons contributes to neuroinflammation, activation of NF-κB, inactive transcriptional expression of IκB-α promoter, and neuropathic pain in oxaliplatin-treated rats. However, as the spinal dorsal horn receives input from the dorsal root ganglia neurons and these degenerate in CIPN, it is still possible that MT2 in the dorsal root ganglia neurons has a critical role in the development of CIPN. Future studies will need to include the MT2 alterations in the dorsal root ganglion neurons and distal nerve endings to parse out any potential peripheral implication in CIPN. Moreover, open field is a rough analysis of overall locomotor activity as it only shows you how much the animal moves but not how well. RotaRod, staircase test, pole test, etc. would better test the locomotor function. But as oxaliplatin-induced neuropathy is a predominantly sensory neuropathy, locomotor activity is not the most important readout parameter.

## Conclusions

The data presented here suggest a central etiological contribution of neuronal MT2 signaling in painful CIPN and provide insight into the neuronal MT2-driven NF-κB pathway inhibition mechanisms induced by the administration of oxaliplatin. It is known that targeting MT2 does not interfere with the anti-cancer mechanisms of chemotherapeutics. Therapeutic efforts centered on the development of MT2 agonists as an adjuvant to oxaliplatin are thus warranted for the prevention and treatment of painful CIPN in human patients.

## Data Availability

The data used and/or analyzed during the current study are available from the corresponding authors on reasonable request.

## Abbreviations

MT2: Metallothionein-2
CIPN: Chemotherapy-induced peripheral neuropathy
CNS: Central nervous system
DRG: Dorsal root ganglion
Co-IP: Coimmunoprecipitation
TSS: Transcription start site
